# The dynamics of nitrogen derived from a chemical nitrogen fertilizer with treated swine slurry in paddy soil-plant systems

**DOI:** 10.1371/journal.pone.0174747

**Published:** 2017-03-24

**Authors:** Joonhee Lee, Hong L. Choi

**Affiliations:** 1 Department of Agricultural Biotechnology, Seoul National University, Seoul, Republic of Korea; 2 Research Institute of Agriculture and Life Sciences, Seoul, Republic of Korea; University of Delhi, INDIA

## Abstract

A well-managed chemical nitrogen (N) fertilization practice combined with treated swine slurry (TSS) is necessary to improve sustainability and N use efficiency in rice farming. However, little is known about the fate of N derived from chemical N fertilizer with and without TSS in paddy soil-plant systems. The objectives of this study were (1) to estimate the contribution of applied N fertilizer to N turnover in rice paddy soil with different N fertilization practices that were manipulated by the quantity of treated swine slurry and chemical N fertilizer (i.e., HTSS+LAS, a high amount of TSS with a low amount of ammonium sulfate; LTSS+HAS, a low amount of TSS with a high amount of ammonium sulfate; AS, ammonium sulfate with phosphorus and potassium; C, the control) and (2) to compare the rice response to applied N derived from each N fertilization practice. Rice biomass yield, ^15^N recovery in both rice grain and stems, soil total N (TN), soil inorganic N, and soil ^15^N recovery were analyzed. Similar amounts of ^15^N uptake by rice in the TSS+AS plots were obtained, indicating that the effects of the different quantities of TSS on chemical fertilizer N recovery in rice during the experimental period were not significant. The soil ^15^N recoveries of HTSS+LAS, LTSS+HAS, and AS in each soil layer were not significantly different. For the HTSS+LAS, LTSS+HAS and AS applications, total ^15^N recoveries were 42%, 43% and 54%, respectively. Because the effects of reducing the use of chemical N fertilizer were attributed to enhancing soil quality and cost-effectiveness, HTSS+LAS could be an appropriate N fertilization practice for improving the long-term sustainability of paddy soil-plant systems. However, N losses, especially through the coupled nitrification-denitrification process, can diminish the benefits that HTSS+LAS offers.

## Introduction

Rice grain yield has increased with the rapid increase in the use of inorganic N fertilizers, especially the ammonium forms of N fertilizers. The application of ammonium-based N fertilizer to irrigated rice paddy soil is the generally accepted method for avoiding denitrification losses. However, the indiscriminate use of chemical N fertilizers causes the long-term depletion of soil organic matter [1]. Thus, the application of organic amendments (such as livestock manure) with chemical N fertilizer has been used as an alternative for chemical N fertilization and related long-term based studies have also been conducted [2–8]. Livestock manure generated from swine houses is a good source of organic amendment in South Korea because of its local abundance and high productivity. According to the Korean Statistical Information Service in 2016, approximately 10 million pigs were raised through concentrated animal feeding operations (CAFOs) [9]. The number of CAFOs have been increasing throughout the country, particularly in local areas in South Korea. The high amount of swine slurry production has always been an inevitable byproduct of CAFOs. Most local governments in South Korea are not allowed to build or expand pig farms due to the farm odor. These circumstances are favorable for large-scale pig farms that can operate expensive odor reduction facilities and swine slurry treatment systems. Thus, small-scale farms with less than 1,000 pigs are typically unable to control odor complaints and treat pig manure and tend to abandon their farm operations. The biological treatment of swine slurry is essential before it flows into soil-water systems because of its high organic matter (OM) and nutrient contents, which could cause serious environmental problems. Air and water pollution have been caused by excessive livestock manure nutrients [10, 11]. The high amount of manure produced by confined livestock in restricted areas has become a serious environmental concern because excess nutrients can adversely affect both the ground water and surface water via leaching and runoff, respectively [12]. These environmental issues have been highlighted, and a movement towards an environmentally friendly treatment system for swine slurry has been initiated. Currently, anaerobic digestion coupled with an aerobic/anoxic reactor is considered a more sustainable swine slurry treatment system to produce treated swine slurry (TSS). Although anaerobic digestion has been suggested as an effective solution to treat swine slurry, the anaerobically digested slurry or sludge remaining in the reactor remains as another negative environmental influence. Previous studies have shown that anaerobically digested slurry applied as an N source in paddy fields and grasslands had a negative impact on the environment, occurring primarily through ammonia (NH_3_) and nitrous oxide (N_2_O) volatilization [13, 14]. Additionally, Bernal and Kirchmann [15] reported in their incubation experiments that NH_3_ volatilization after 9 days of application of anaerobically treated pig manure was higher than that of aerobically treated pig manure. Thus, the combined anaerobic-aerobic approach is being implemented as an alternative to the conventional anaerobic digestion processes because it can effectively remove OM and N that are present in anaerobically digested slurry [16, 17]. However, little is known about the mechanisms of a land application of chemical N fertilizer with TSS, especially the fate of N derived from chemical N fertilizer in a paddy soil-plant system when TSS is also used. Accordingly, the objectives of this study were (1) to estimate the contribution of applied N fertilizer to N turnover in a rice paddy soil with different N fertilization practices that are manipulated by the quantity of TSS and chemical N fertilizer and (2) to compare the rice response to applied N derived from each N fertilization practice.

## Materials and methods

### Lysimeter description

Twelve lysimeters (250 mm in diameter and 500 mm in depth) were prepared using a PVC pipe and installed in a greenhouse (15.0 m × 9.8 m) that was located at a livestock experimental farm in Suwon, South Korea. Each lysimeter contained an undisturbed soil monolith, which was extracted from an agricultural field located near Suwon (37°15’51”N, 126°58’48”W) with the permission of University Animal Farm, College of Agriculture & Life Science, Seoul National University. The soil used in this study was a coarse loamy, mixed, mesic family of Typic Dystrudepts. The amount of OM in the soil sample was 9.0 g kg^-1^. The baseline soil sample had a mean pH of 5.8 (1:5 water). The available inorganic elements in the baseline soil sample include 299 mg kg^-1^ of phosphorous [18], 0.59 cmol^+^kg^-1^ of potassium ion (K^+^), 4.7 cmol^+^kg^-1^ of calcium ion (Ca^2+^), and 1.5 cmol^+^kg^-1^ of magnesium ion (Mg^2+^) (ICP-MS analysis). The electrical conductivity (EC) was 0.4 dS/m. The TN, NO_3_^-^, and NH_4_^+^ levels of the initial soil were 0.14%, 9.48 mg kg^-1^, and 9.67 mg kg^-1^, respectively. The leachate drained through the perforated bottom plate of the lysimeter to mimic natural leaching conditions. Rice (*Oryza sativa* L.) seedlings were grown in a seedling box (30 cm × 60 cm) and transplanted to the lysimeters on July 5, 2014. The rice was harvested on 24 December 2014. Rice biomass at 5 cm above the soil surface was harvested and placed into a paper bag. The paper bags containing the rice biomass were oven dried (35°C for 10 days), and the weight was measured. The dried rice was separated into the grain and stem portions, ground in a stainless-steel mill and sieved through a 1.0-mm screen.

### Experimental design

**Fig 1:**
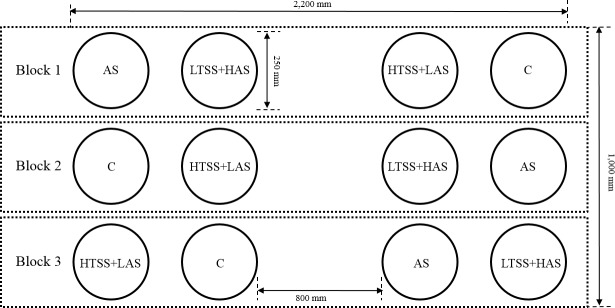
Experimental layout. A randomized complete block design; the main plots consisted of three replicates. The main plots were divided into four subplots corresponding to the four treatments (HTSS+LAS, LTSS+HAS, AS, and C).

**Fig 2:**
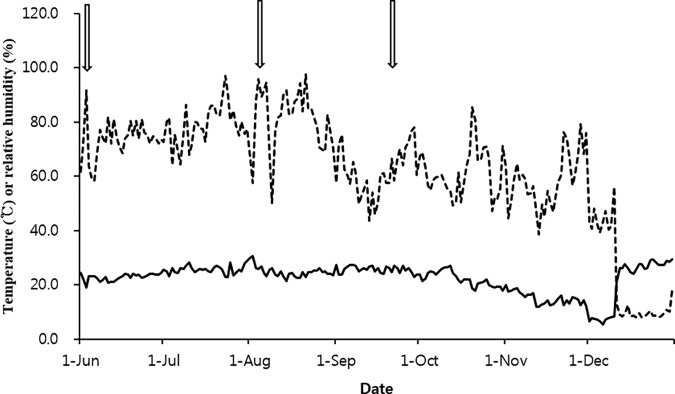
Relative humidity (dashed-line) and temperatures (solid-line) at the livestock experimental farm in Suwon, South Korea during the 2014 experimental period. The open arrow indicates the rainfall date using a simulator. To prevent the rainfall simulator from freezing, a heater was used at the experimental site on December 11.

A randomized complete block design was used in the study in which the main plots consisted of three replicates (Fig 1). The main plots were divided into four subplots corresponding to the four treatments: a high amount of treated swine slurry with a low amount of ^15^N-labeled ammonium sulfate (HTSS+LAS), a low amount of treated swine slurry with a high amount of ^15^N-labeled ammonium sulfate (LTSS+HAS), ^15^N-labeled ammonium sulfate with phosphorus and potassium (AS), and a control (C, no soil amendments). Each treatment was evenly distributed on the soil surface in the lysimeter (0.073 m^2^) using different colored stickers to distinguish each subplot. The temperature and humidity data were collected at a weather station (WatchDog 2900ET, Spectrum Technologies, Inc.) at the experimental site (Fig 2). The source water for the simulation was tap water, which was stored in a 1.0 m^3^ tank for at least two weeks before rainfall to remove hypochlorite. Three rainfall events (June 03, August 10 and September 21) with moderate intensity (9.0 mm hr^-1^) were applied during the experimental period. The surface water level of each plot was maintained at 2 cm above the soil surface through rainfall and irrigation until September 30 to exemplify a waterlogged paddy plot.

### Application of chemical N fertilizer (^15^N-labelled ammonium sulfate) with treated swine slurry

Swine slurry was produced from a livestock experimental farm in Suwon, South Korea and was treated using a biological wastewater treatment (BWWT) system. The system was constructed as a pilot scale (1,000 L of swine slurry/day) and consisted of sequencing the reactors using activated sludge processes. First, the raw swine slurry goes through an underground anaerobic digestion (UGAD, 20 m^3^ capacity) process as a pretreatment to produce biogas (methane) and to reduce the high concentration of OM in the swine slurry. Then, the anaerobically digested swine slurry flows into the BWWT reactors that consist of five aerobic-anaerobic tanks (Tank 1 and Tank 4 are anaerobic processes; Tank 2, Tank 3, and Tank 5 are aerobic processes). A 10-kg slurry sample collected from Tank 5, where the OM, N, and phosphorus were reduced, was transferred to a 20-L pail. TSS was well mixed and divided into 1-L plastic sample bags for eventual lysimeter application. The TSS sample bags were frozen as quickly as possible to prevent NH_3_ gas losses and stored at -15°C. The total solids (TS), volatile solids (VS), total chemical oxygen demand (TCOD), TN, and pH of TSS were 15.6 g L^-1^, 8.6 g L^-1^, 13.1 g L^-1^, 0.65 g L^-1^ and 5.16, respectively. Rice was fertilized with TSS and labeled with ^15^N AS (5 atom % ^15^N) at an equivalent rate of 80 kg N ha^-1^ (60 kg N ha^-1^ TSS and 20 kg N ha^-1^ AS for the HTSS+LAS treatment and 26.7 kg N ha^-1^ TSS and 53.3 kg N ha^-1^ AS for the LTSS+HAS treatment). All treatments (HTSS+LAS, LTSS+HAS, and AS) were applied as split applications (three-fourths on August 08 and one-fourth on September 19). The rates of the TN applied in HTSS+LAS, LTSS+HAS, and AS are given in Table 1.

**Table 1:** List of treatments and the application rate of nitrogen (N).

Treatments		Rate of TN application (kg ha^-1^)
**HTSS+LAS**	Treated swine slurry and ammonium sulfate (3:1 ratio)	80 ^a^
**LTSS+HAS**	Treated swine slurry and ammonium sulfate (1:2 ratio)	80 ^a^
**AS** ^b^	Ammonium sulfate only	80 ^a^
**C**	Control	No fertilizer, treated swine slurry applied

^a^ Split application is used (applied two-thirds of TN at the tillering stage and one-third of TN at panicle initiation).

^b^ AS treatment includes phosphorus (45 kg ha^-1^) and potassium (57 kg ha^-1^).

### Chemical analysis

The TS and VS values of TSS were analyzed according to APHA standard methods. The TCOD and TN of TSS was determined via the Hach chemical reagents manual (DR 5000, Hach, Loveland, Colo.). A stainless steel auger (12.6-mm diameter) was used to collect the soil sample. Ten soil cores of each soil depth (0–5, 5–10, 10–20 and 20–30 cm) were collected after rice harvest in December 2014, composited, and placed in plastic bags for TN, NO_3_^-^, NH_4_^+^, and ^15^N analysis. The soil samples were mixed well in the bag, and 5.0 g was weighed and then dried at 105°C for 24 hours. The atom % ^15^N and TN values of the dried soil samples were analyzed via the combustion method using an elemental analyzer linked to a stable isotope mass spectrometer (Isoprime-EA, Micromass, UK). The isotopic ratios were reported in standard notations with respect to atmospheric N gas (N_2_) as the working standard (Ozteck, USA; δ^15^N value of– 0.22‰). The accuracy (<1.0‰) and reproducibility (<0.5‰) of the measurements were verified using a reference material (IAEA-N3, KNO_3_) from the International Atomic Energy Agency. Ten grams of soil and 100 mL of 2 M KCl were added to 250-mL plastic bottles and shaken for 30 min. After centrifuging for 5 minutes, the KCl extract was filtered through Whatman no.1 filter paper. Devarda’s alloy (0.5 g) and MgO (0.5 g) were added to the 50 mL of subsample and analyzed for NH_4_^+^_N and NO_3_^-^_N using a Kjeltec auto 2400/8400 System (Tecator AB, Sweden).

### Rice ^15^N uptake, soil ^15^N recovery and total ^15^N recovery calculation

The recovery of AS ^15^N in harvested rice and soil was calculated using the procedures outlined by Powell et al. [19]. Rice ^15^N uptake was calculated according to Eq (1).

$$Rice\;15N\;recov\;\%=\frac{P\;(c-d)}{f\;(a-b)}\times 100$$ (1)

In this equation, *P* is total rice N uptake (mg), *f* is ammonium sulfate N (mg), *a* is the atom % ^15^N of the applied ammonium sulfate, *b* is the atom % ^15^N in non-labeled ammonium sulfate (assuming 0.366 for all three inputs), *c* is the atom % ^15^N in rice (an average of 3 circles within a treatment), and *d* is the atom % ^15^N in the control rice (an average of 3 ‘control’ circles). The recovery of applied ^15^N in total soil N was calculated according to Eq (2).

$$Soil\;15N\;recov\;\%=\frac{Q\;(e-g)}{f\;(a-b)}\times 100$$ (2)

where *Q* is total soil N, *e* is the atom % ^15^N of total soil N in the treatment plots, *g* is the atom % ^15^N in the control plots, and the other terms are identical to those described for Eq (1). The total ^15^N recovery was calculated as the sum of the recoveries in the rice and soil components using Eq (3).

$$Total\;N\;recovery\;\%={\%N\;recov}_{harv\;rice}+{\%N\;recov}_{soil}$$ (3)

### Statistical analysis

The differences in rice ^15^N uptake and soil ^15^N recovery between soil depths and the different N fertilization practices were analyzed using ANOVA with IBM SPSS STATISTICS 22. Tukey’s test was used for post-hoc analysis. The correlation between ^15^N isotope abundance (δ scale, the dependent variable) and inorganic N (independent variable) in soil was analyzed using the linear regression analysis model. The analysis of variance was conducted at a significance level of 0.05.

## Results

### Dry matter yield and total N uptake by rice

**Fig 3:**
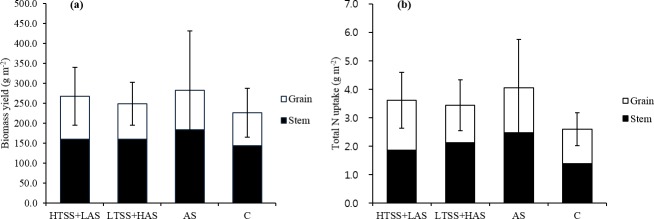
**Biomass (dry matter) yield (a) and Total N uptake by rice after application of treated swine slurry and chemical fertilizer (b).** Error bars represent standard deviations (n = 3) of the means of rice biomass yield (combined value of grain and stem) and total N uptake by rice (combined value of grain and stem).

The effects of HTSS+LAS, LTSS+HAS, and AS on rice biomass production and rice TN uptake are shown in Fig 3. The highest biomass yield (282.2 g m^-2^) and the lowest biomass yield (248.7 g m^-2^) were obtained for the AS application and the LTSS+HAS application, respectively. The total biomass yield increased by 18% and 10% under HTSS+LAS and LTSS+HAS compared with the control plot, respectively. Similarly, the TN uptake by rice for the AS application (4.06 g m^-2^) was higher than the HTSS+LAS (3.62 g m^-2^) and LTSS+HAS (3.44 g m^-2^) applications. The TN uptake by rice decreased by 11% and 15% under HTSS+LAS and LTSS+HAS compared with the AS plot, respectively. However, there was no significant effect on biomass production (whole plant) and rice TN uptake between the treatments. The AS application had a relatively large standard deviation for biomass yield and TN uptake, which was probably due to the inhomogeneous sunlight distribution in the greenhouse.

### Soil total nitrogen, soil nitrate N, ammonium N and total soil ^15^N recovery

Soil TN, soil nitrate N (NO_3_^-^), ammonium N (NH_4_^+^), and total soil ^15^N recovery after application of HTSS+LAS, LTSS+HAS and AS are shown in Table 2. The different N fertilization practices did not affect the soil TN. A significant decrease in soil TN was observed with an increase in soil depth, regardless of fertilization practice. Regarding soil NO_3_^-^, there was a significant difference between the treatments, especially for the HTSS+LAS and LTSS+HAS applications in the upper soil layer (0–5 cm). HTSS+LAS and AS had a similar effect on the retention of soil NO_3_^-^ in the same soil layer. A general reduction in soil NO_3_^-^ was observed until the soil depth of 30 cm, indicating that NO_3_^-^ is less available in deeper soil. However, in the AS application, the tendency towards a steady increase was observed after the 20-cm soil depth. The effect of fertilization practice on soil NH_4_^+^ was not clear at each soil depth, except at the depth of 10–20 cm. The amount of NH_4_^+^ in the soil decreased with depth regardless of the fertilization practice. The soil ^15^N recoveries of HTSS+LAS, LTSS+HAS, and AS in each soil layer were not significantly different. Over 70% of the soil ^15^N recovery was observed in the upper two soil layers (0–5 and 5–10 cm) after the application of each treatment. The predicted influence of the inorganic N form (NO_3_^-^ and NH_4_^+^) on δ ^15^N in different soil layers is shown in Table 3. The N forms NO_3_^-^ and NH_4_^+^ had positive and negative associations with soil δ ^15^N in the top 10 cm and 10–20 cm of the soil, respectively.

**Table 2:** Soil total nitrogen (TN), soil nitrate N (NO_3_^-^), ammonium N (NH_4_^+^), and total soil ^15^N recovery following an application of ^15^N-labeled ammonium sulfate and treated swine slurry.

	Treatments			
	Soil depth (cm)	HTSS+LAS	LTSS+HAS	AS
**Total N (g kg**^**-1**^**)**	0–5	1.9 ns(a)	1.9 ns(a)	2.0 ns(a)
	5–10	1.6 ns(ab)	1.7 ns(ab)	1.6 ns(b)
	10–20	1.2 ns(bc)	1.2 ns(bc)	1.3 ns(c)
	20–30	0.8 ns(c)	0.9 ns(c)	0.9 ns(d)
**Nitrate N (mg kg**^**-1**^**)**	0–5	28.3 A(a)	17.3 B(a)	26.1 A(a)
	5–10	13.7 ns(b)	12.8 ns(ab)	16.7 ns(ab)
	10–20	9.3 ns(bc)	10.9 ns(b)	11.3 ns(b)
	20–30	3.4 B(c)	8.8 AB(b)	15.2 A(ab)
**Ammonium N (mg kg**^**-1**^**)**	0–5	16.8 A(a)	14.5 A(a)	14.5 A(a)
	5–10	12.4 ns(b)	12.6 ns(a)	11.0 ns(b)
	10–20	9.4 A(c)	9.8 A(b)	7.7 B(c)
	20–30	5.4 ns(d)	6.0 ns(c)	6.2 ns(c)
**Soil** ^**15**^**N recovery (%)**	0–5	21.1 ns(a)	20.1 ns(a)	22.2 ns(a)
	5–10	5.0 ns(b)	8.3 ns(b)	6.7 ns(b)
	10–20	4.3 ns(bc)	4.3 ns(b)	7.4 ns(b)
	20–30	2.7 ns(c)	3.2 ns(b)	1.8 ns(b)

Data with the different capital letters and small letters in the parentheses indicate significant differences (p-value < 0.05) among the treatments (row) and soil depth (column), respectively.

ns represents not significant (p-value > 0.05).

**Table 3:** Linear regression analysis used to describe the predicted influence of the inorganic N form on δ ^15^N in different soil layers.

Soil depth (cm)	Independent variables	B	β	t	P
**0–10**	Nitrate N (NO_3_^-^)	9.544	0.909	3.537	*
	Ammonium N (NH_4_^+^)	-11.807	-0.364	-1.417	ns
**10–20**	Nitrate N (NO_3_^-^)	2.802	0.223	1.741	ns
	Ammonium N (NH_4_^+^)	-17.098	-0.919.	-7.159	**
**20–30**	Nitrate N (NO_3_^-^)	0.001	0.001	0.001	ns
	Ammonium N (NH_4_^+^)	4.926	0.354	0.633	ns

Dependent variable is soil δ ^15^N (‰); B, unstandardized coefficients; β, beta-value; t, t-value; P, p-value for the independent variable (p-value < 0.05).

*p-value < 0.01.

**p-value < 0.001; ns represents not significant (p-value > 0.05).

### Nitrogen-15 recoveries in the soil-plant system

Nitrogen-15 recoveries of the labeled N source with the different types of N fertilization practice (HTSS+LAS, LTSS+HAS, and AS) in the soil-plant system are shown in Table 4. In the case of TN uptake by rice, it is possible that some N from TSS and AS, as well as from soil N, affected TN. However, rice ^15^N recovery only represents the amount recovered from applied chemical fertilizer. For rice ^15^N recovery, the HTSS+LAS amended plots showed larger ^15^N recovery in the rice grain (4.8%) than in the stem (3.8%). In contrast, 4.8% and 8.5% of the total applied ^15^N, which is larger than the recovery in grain, were recovered from the rice stems following the LTSS+HAS and AS treatments, respectively. In the case of rice ^15^N recovery (whole plant), there was a significant recovery difference (p-value < 0.05) between the AS and other different N fertilization practices (HTSS+LAS and LTSS+HAS). Rice ^15^N uptake with LTSS+HAS was 13% and 51% lower than that of HTSS+LAS and AS, respectively. In addition, the AS application resulted in greater soil ^15^N recovery (38%) compared with the other N sources. The recovery of ^15^N in the HTSS+LAS amended plots was lower (33%) than that in the recovery of the LTSS+HAS amended plot (36%). For the HTSS+LAS, LTSS+HAS and AS applications, the total ^15^N recoveries were 42%, 43% and 54%, respectively. A larger amount of unaccounted ^15^N was observed in the HTSS+LAS treatment (58%) compared to the other fertilizer N sources.

**Table 4:** Nitrogen-15 recoveries of the labeled N source (ammonium sulfate) in the different fertilization practices in a paddy soil-plant system.

Treatment	Grain ^15^N recovery (%)	Stem ^15^N recovery (%)	Total rice ^15^N recovery (%)	Soil ^15^N recovery (%)	Total ^15^N recovery (%)	Unaccounted for ^15^N (%)
**HTSS+LAS**	4.8 ab	3.8 ns	8.6 a	33.1	41.7	58.3
**LTSS +HAS**	2.7 a	4.8 ns	7.5 a	35.9	43.4	56.6
**AS**	6.8 b	8.5 ns	15.3 b	38.2	53.5	46.5

Data with different letters in the same column are significantly different (p-value <0.05).

ns represents not significant (p-value > 0.05).

## Discussion

### Rice response to the applied N derived from each N fertilization practice

The application of HTSS+LAS, LTSS+HAS and AS resulted in similar rice biomass yield (whole plant) and TN uptake mainly due to the equal TN application rate (80 kg ha^-1^) of the treatments. However, considering cost-effectiveness, the application of HTSS+LAS on rice fields is recommended as the N fertilization practice. The difference of chemical fertilizer N amount from each treatment is as follows. AS, LTSS + HAS and HTSS + LAS had a chemical fertilizer N content of 80 kg ha^-1^, 53 kg ha^-1^ and 20 kg ha^-1^, with the corresponding ratio of 1: 0.67: 0.25, respectively. Chemical fertilizer N is characterized by a fast-release effect that can be absorbed instantaneously by rice after N is applied. Therefore, AS has relatively high rice yield and TN uptake. TSS used as organic amendment also had a partial fast-release effect and showed no significant differences in biomass yield and TN uptake compared to the AS treatment. Although rice yield and TN uptake responses to the different N fertilization practices were not significantly different, rice ^15^N uptake following AS deposition was significantly higher than that observed for the HTSS+LAS and LTSS+HAS treatments. The chemical fertilizer used with TSS showed similar rice N recoveries of 8.6% and 7.5% for HTSS + LAS and LTSS + HAS, respectively. On the other hand, the N recovery of AS was 15.3%, which was more than 6% higher than that of the mixed treatment. This indicated that the organic amendment and the native soil N showed a potential to contribute to TN recovery by 43% in HTSS and 51% in LTSS, respectively. In the present study, when TSS was three times that of AS, the chemical fertilizer N uptake increased more than the TSS. On the other hand, when AS was twice that of TSS, chemical fertilizer N was used less than the TSS N. This is most likely explained by the fact that the uptake of chemical fertilizer N seems to be improved in the HTSS+LAS treatment due to increased soil microbial activity [3, 8, 20]. The positive effects of the combined HTSS and LAS on the rice N recovery was observed, especially in rice grain N recovery. Similarly, Lee et al. [21] reported that a dairy slurry application did not affect the whole plant barley N yield, but there was an N response in the barley kernel with the dairy manure application. The high protein level in rice grain associated with the overuse of N fertilizer has a negative impact on the cooking and eating quality of the grain due to low gel consistency, gelatinization or amylose content [22, 23]. Given this fact, the use of AS fertilizer as a source of N may be avoided in terms of rice grain quality.

### The contribution of applied N fertilizer to N turnover in the rice paddy soil with different N fertilization practices

Most of the applied ^15^N was recovered from the top 10 cm of all N amended soil, which was in agreement with a previous study that suggested that very little applied ^15^N was recovered in the soil, except that from the surface organic layer [24]. According to the correlation between soil inorganic N (NO_3_^-^ and NH_4_^+^) and δ^15^N values after fertilizer applications, the NO_3_^-^ N form in the soil had a larger positive beta value (β = 0.909) than that of the NH_4_^+^ N form in the upper two soil layers (Table 3). This can be understood in terms of the nitrification processes that occur in the surface paddy soil regardless of the different N treatments. As the NH_4_^+^ is converted to NO_3_^-^ through nitrification in the oxidized paddy soil surface, an increase of soil δ ^15^N is obtained because ^15^N-depleted NH_4_^+^ is decreased [25]. The N fertilizer (HTSS+LAS, LTSS+HAS and AS) deposited in each plot under waterlogged conditions may have resulted in a limited nitrification process immediately after the application, except for the paddy soil surface, causing certain soil bacteria to obtain oxygen (O_2_) from NO_3_^-^. With the reduction of produced NO_3_^-^ progresses in the lower anaerobic soil layers, NO_3_^-^ is serially converted to nitrite (NO_2_^-^), nitric oxide (NO), N_2_O, and N_2_ gas [26, 27]. Consequently, relatively lower δ^15^N soil NH_4_^+^ and higher δ^15^N soil NO_3_^-^ were observed in waterlogged paddy soil due to restricted nitrification in saturated soil conditions [28]. The N form of NH_4_^+^ has a negative correlation with soil δ ^15^N in the 10–20 cm soil layer. In other words, a decrease of soil δ ^15^N would be observed as NH_4_^+^ is converted to NO_3_^-^ through nitrification in the rhizosphere of the paddy soil. This may be explained by the results of Arth and Frenzel [29], who observed that a substantial amount of O_2_ is measured around the rice roots, which provides aerobic conditions within the anoxic soil layer, especially during the rice growing period [30]. A laboratory incubation study conducted by Mohanty et al. [31] reported that fertilization practice (a mixture of farmyard manure and chemical fertilizer) and aerobic soil conditions resulted in relatively high N mineralization kinetics in the soil compared with that of a single fertilizer and anaerobic conditions. After the waterlogged period, some of the NH_4_^+^ transformed by the microbes in TSS amended soil is adsorbed onto negatively charged clay particles, and the plant roots absorb some as well. The NH_4_^+^ adsorbed onto clay particles is converted to NO_3_^-^ via nitrification. However, the undigested N that is passed through the biological treatment system without being used is decomposed slower in the soil compared with inorganic N. Thus, the mineralization rate of AS is the largest, followed by the less rapid mineralization of TSS undigested N.

### The fate of ^15^N derived from a chemical N fertilizer with TSS in paddy soil-plant systems

The result of the total ^15^N recovery was somewhat smaller (approximately from 42% to 54%) than the value reported by Zhao et al. [32]. The study investigating the fate of fertilizer ^15^N in paddy soil using an undisturbed monolith lysimeter showed that the ^15^N recovery in rice and soil were 41% and 22%, respectively. The amount of ^15^N recovery using the ^15^N direct methods may be low because the amount of available ^15^N caused by pool substitution would not be used in the recovery calculations [33]. The interaction between the added N and native soil N has been described in previous studies [33, 34]. Jenkinson et al. [34] introduced the term added N interaction (ANI), which is used to indicate that the added N fertilizer can increase the mineralization of the unlabeled soil N. Additionally, the low ^15^N recovery of applied ^15^N may occur due to uneven fertilizer application and high variability in bulk density. We assumed that the bulk density of the soil ranged from 1.1 to 1.3 g cm^-3^, depending on soil depth. In the present study, the calculated unaccounted ^15^N ranged from 46% to 58%, which is higher than in previous studies. Schnier et al. [35] reported that the total soil and plant ^15^N recoveries ranged from 67% to 78% at the mature stages, and approximately 22% to 33% of applied ^15^N fertilizer could not be accounted for in either N remaining in the soil or via plant N uptake. The N losses through NH_3_ volatilization, leaching and runoff, and direct N_2_O emissions were 12%, 0.3% and 0.12%, respectively [32]. NH_3_ volatilization in this study could be limited because the pH of the AS and TSS amended soils were less than 9.0 (data not shown), which is the pH level where the predominant form of N is NH_4_^+^. Our results showed the potential for the occurrence of a coupled nitrification-denitrification process due to an oxidized soil surface and aerobic soil conditions around the rice roots in the rhizosphere. Therefore, when considering aerobic and anaerobic soil conditions in the rice paddy soil, the coupled nitrification-denitrification process could play a key role in determining N losses in this study [36, 37]. When TSS and AS are used together, a certain amount of AS N tends to be lost, irrespective of the amount of AS used. Therefore, it is a good strategy to use less chemical fertilizer N in terms of soil quality and cost-effectiveness. TSS can be used as a major N source in rice fields, and chemical fertilizer N might be a complement to TSS. Although the results of this study are generally observed in similar environments, there were some potential limitations. In general, N use efficiency related to the use of chemical fertilizers and organic amendments is interpreted by converging the results over a long-term period [3, 5, 38]. The present study is a relatively short period compared to long-term research, only focusing on the period from the vegetative stage to harvest. In addition, the number of treatments (i.e., AS, HTSS+LAS, LTSS+HAS and control) in this study was relatively small. The research should have more treatments at different N levels to generalize the results for an appropriate N fertilization practice. For these reasons, the findings of the present study might not represent the broader rice cropping system based on this study alone. However, the results of the present study could help in determining the chemical fertilizer application rate in the next year through the precise ^15^N analysis when mixed organic amendment (TSS) and chemical fertilizer were used.

## Conclusions

The proper use of N fertilizer is an essential step towards achieving successful rice farming. The application of chemical N fertilizer with organic amendments (such as livestock manure) has been used to improve sustainability and nutrient use efficiency. The TN uptake by rice for the AS, HTSS + LAS, and LTSS + HAS were 4.06 g m^-2^, 3.62 g m^-2^ and 3.44 g m^-2^, respectively. These TN values were partly influenced by the organic fertilizer N, soil N, and chemical fertilizer N. On the other hand, rice ^15^N recovery (whole plant) only represents the amount recovered from applied chemical fertilizer, and there was a significant recovery difference (p <0.05) between the AS and mixed N fertilization practices. Similar amounts of ^15^N uptake by rice in the TSS+AS plots were obtained, indicating that the effects of the different quantities of TSS on chemical fertilizer N recovery in rice during the experimental period were not significant. The soil ^15^N recoveries of HTSS+LAS, LTSS+HAS, and AS in each soil layer (S1 Fig) were not significantly different. Most of the applied ^15^N was recovered in the top 10 cm of all N amended soil. We note that those N compounds may be affected by the nitrification processes occurring in the surface paddy soil regardless of the different N fertilization practices. As NH_4_^+^ is converted to NO_3_^-^ through nitrification in the oxidized paddy soil surface, the increase of soil δ ^15^N would be obtained because ^15^N-depleted NH_4_^+^ decreased. For the HTSS+LAS, LTSS+HAS and AS applications, the total ^15^N recoveries were 42%, 43% and 54%, respectively. Consequently, a relatively larger amount of unaccounted for ^15^N was observed for the HTSS+LAS treatment (58%) compared to the other N fertilization practices. Our results showed that HTSS+LAS has a good potential to improve the long-term sustainability of paddy soil-plant systems because the effects of reducing the use of chemical N fertilizer is attributed to enhancing soil quality and cost-effectiveness. However, N losses, especially through the coupled nitrification-denitrification process, can diminish the benefits that HTSS+LAS offers. A direct measurement of ^15^N loss pathways (i.e., N_2_O and NH_3_ volatilization, leaching and surface runoff, denitrification, and immobilization) is required to estimate an accurate N balance in rice paddy soil-plant systems.

## Supporting information

S1 Fig **Soil** ^**15**^**N recovery (a) and soil δ** ^**15**^**N (b) after an application of treated swine slurry and chemical fertilizer.** Error bars represent standard deviations (n = 3) of the means of soil ^15^N recovery and soil δ ^15^N, respectively.
(TIF)
