# Supplementary figures and images for: The dynamics of nitrogen derived from a chemical nitrogen fertilizer with treated swine slurry in paddy soil-plant systems
Source: PLoS One. 2017 Mar 24;12(3):e0174747. doi: 10.1371/journal.pone.0174747 (PMC5365117; doi:10.1371/journal.pone.0174747)

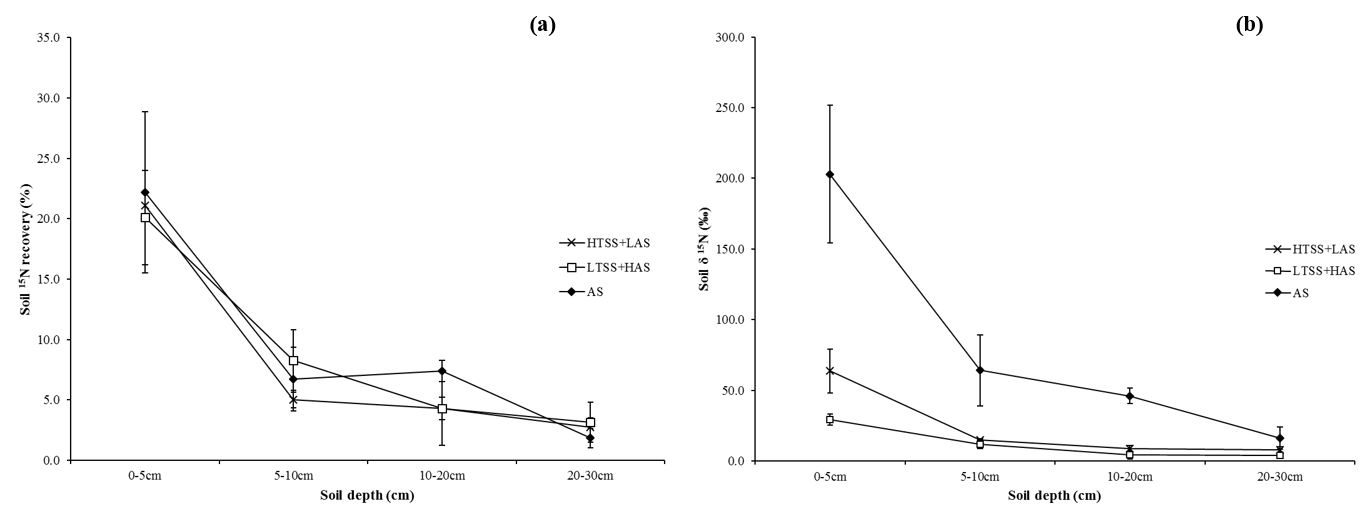

Supplement: S1 Fig — Soil 15N recovery (a) and soil δ 15N (b) after an application of treated swine slurry and chemical fertilizer. Error bars represent standard deviations (n = 3) of the means of soil 15N recovery and soil δ 15N, respectively. (TIF) [file pone.0174747.s001.tif]
